# Non-Focal Neurological Symptoms Associated with Classical Presentations of Transient Ischaemic Attack: Qualitative Analysis of Interviews with Patients

**DOI:** 10.1371/journal.pone.0066351

**Published:** 2013-06-12

**Authors:** Susan Kirkpatrick, Louise Locock, Matthew F. Giles, Daniel S. Lasserson

**Affiliations:** 1 Health Experiences Research Group, Department of Primary Care Health Sciences, University of Oxford, Oxford, United Kingdom; 2 Stroke Prevention Unit, Nuffield Department of Clinical Neuroscience, University of Oxford, Oxford, United Kingdom; 3 Department of Primary Care Health Sciences, University of Oxford, Oxford, United Kingdom; University of Cambridge, United Kingdom

## Abstract

**Background:**

Improving the recognition of transient ischaemic attack (TIA) at initial healthcare contact is essential as urgent specialist assessment and treatment reduces stroke risk. Accurate TIA detection could be achieved with clinical prediction rules but none have been validated in primary care. An alternative approach using qualitative analysis of patients' experiences of TIA may identify novel features of the TIA phenotype that are not detected routinely, as such techniques have revealed novel early features of other important conditions such as meningococcaemia. We sought to determine whether the patient's experience of TIA would reveal additional deficits that can be tested prospectively in cohort studies to determine their additional diagnostic and prognostic utility at the first healthcare contact.

**Methodology and Findings:**

Qualitative semi-structured interviews with 25 patients who had experienced definite TIA as determined by a stroke specialist; framework analysis to map symptoms and key words or descriptive phrases used against each individual, with close attention to the detail of the language used. All interview transcripts were reviewed by a specialist clinician with experience in TIA/minor stroke. Patients described non-focal symptoms consistent with higher function deficits in spatial perception and awareness of deficit, as well as feelings of disconnection with their immediate surroundings. Of the classical features, weakness and speech disturbance were described in ways that did not meet the readily recognisable phenotype.

**Conclusion/Significance:**

Analysis of patients' narrative accounts reveals a set of overlooked features of the experience of TIA which may provide additional diagnostic utility so that providers of first contact healthcare can recognise TIA more easily. Future research is required in a prospective cohort of patients presenting with transient neurological symptoms to determine how frequent these features are, what they add to diagnostic information and whether they can refine measures to predict stroke risk.

## Introduction

There is a high risk of stroke within the first few days after transient ischaemic attack (TIA) [1] which can be reduced substantially after urgent specialist assessment and treatment [2], [3]. Accurate recognition of TIA at the first healthcare contact is therefore crucial in reducing early stroke risk. Patients with TIA initially seek healthcare from primary care practices and emergency departments [4] and although the relative use of these two sources of first contact healthcare varies by country [5], accurate detection of TIA in both these settings is needed. There are key investigations that stratify stroke risk, such as brain imaging [6], but the diagnosis remains essentially clinical and relies on the report of the episode by the patient with any available witness account.

Detection of TIA therefore relies heavily on the clinical history, which is a largely interrogative process [7]. Nevertheless, there is evidence to suggest that this is a difficult and complex task for primary care physicians– around 50% of referrals to TIA clinics do not have a cerebrovascular diagnosis [8] and vignette studies have demonstrated variation in the ability of primary care physicians to suspect TIA from simple clinical details [9]–[11].

Improving the recognition of TIA is therefore crucial for stroke prevention and whilst there are simple clinical approaches such as clinical prediction rules, which prompt the clinician to ask certain questions that form components of a scoring system [12], this may be limited for TIA recognition. Although a number of stroke recognition tools exist such as the Recognition of Stroke in the Emergency Room (ROSIER) [13] and the Face, Arm and Speech test (FAST) [14], they are based on the demonstration of physical deficit which is unlikely to be present in TIA, particularly in patients presenting to primary care where there is often significant delay between symptom onset and initial assessment [4]. There is only one report of a recognition tool specifically for TIA which is based on the physical signs elicited from patients with stroke [15], rather than from patients' descriptions of their experience of TIA.

An alternative approach to address the evidence gap of improving recognition of TIA is to use qualitative data to reveal novel or previously unrecognised clinical features that are associated with TIA. Qualitative analysis of interviews with patients can mine the experience of transient neurological events to uncover clinical features that may not be elicited through standard medical history taking. Although qualitative methodologies are not routinely used in diagnostic research, these methods have yielded insights which can aid the diagnostic process, for example interviews with parents of children with meningococcaemia [16] have shown that there are novel clinical features that are not routinely elicited in clinical history taking. Furthermore, these novel features are prognostic and have recently changed national guidance in the UK for suspicion of meningococcaemia in primary care [17].

Quantitative methods are not always appropriate to investigate novel or unrecognised clinical associations due to reliance on questionnaires requiring dichotomous data collection (i.e. feature present or absent) as this will require pre-specified questions. Furthermore, data collection methods which briefly ask for several lines of clinical narrative about the symptoms may also not reveal novel or unrecognised features. A qualitative approach with semi-structured interviews allows patients more time to explore and recount the narratives of their episode of transient neurological deficit and novel or unrecognised clinical features can be sought for in the qualitative analysis of interview transcripts. Whilst definitive prospective studies using quantitative methods will always be required to ascertain diagnostic and prognostic significance of clinical features, the qualitative approach is an initial step to investigate the presence of novel features which can then be tested in subsequent cohort studies.

Therefore we undertook semi-structured qualitative interviews with patients with TIA and their carers to gain additional information about the experience of their event. Patients bring to the consultation their ‘embodied knowledge’– internal sensations and observations which may be hidden to the physician, or which may not fit with current diagnostic criteria and are therefore overlooked, even if mentioned by the patient [18].

We restricted the analysis to patients with a diagnosis of definite, rather than possible TIA in order to increase the likelihood that novel or unrecognised descriptors of deficit are not due to inclusion of patients without a cerebrovascular cause for their symptoms.

## Methods

### Sampling and recruitment

The data is drawn from a wider qualitative interview study in the UK using a maximum variation sampling approach. This is designed to capture the broadest possible range of experiences [19]. Variation was sought across demographic variables and types of TIA experience (e.g. one or several TIAs; different types of presentation; different types of referral route). Participants were recruited from a number of sources including the Oxford Vascular study (OXVASC), primary care practices, TIA clinics, support organisations and community groups, and media advertising. The Primary Care Clinical Studies Group of the UK National Institute for Health Research Stroke Research Network acted as expert advisory group to the study. Participants gave written informed consent to be interviewed, and after checking their interview transcript signed a further form giving permission for their interview to be used for teaching, research, publication and online dissemination on the patient information website www.healthtalkonline.org (see below). Ethics approval for the consent procedure and research methods was granted by Berkshire Research Ethics Committee (reference number 91H050516).

### Data collection

37 people (including some family members) were interviewed in 2010 across the UK, usually in the participant's own home. Interviews lasted between one and two hours and were video or audio recorded. They comprised two sections; an unstructured narrative (‘tell me your story’) followed by semi structured prompting about their experiences of diagnosis, treatment and life after TIA. Anonymised data from the study (video and audio interview extracts) are freely available at http://www.healthtalkonline.org/Nerves_and_brain/transient_ischaemic_attack,

As part of their opening narrative, all participants described what their symptoms were like and what made them think something was wrong. Family members were excluded from this analysis.

### Clinical review of transcripts

Because of the range of recruitment sources, the sample included some people with a clinically confirmed diagnosis (notably those recruited through OXVASC), but also others (for example recruited through support groups) where we were reliant on self-report. To overcome this, these interview transcripts were reviewed by a TIA specialist clinician (MG) and participants whose diagnosis was not clinically definite TIA were excluded from the analysis.

### Analysis

Two researchers (SK, LL) re-read and discussed sections of the interviews where symptoms were described. Using framework analysis [20] an initial chart was drawn up mapping symptoms and key words used against each individual. Findings were then discussed with authors MG and DL. Analysis focused on both the vivid and nuanced descriptions given of classic TIA symptoms, as well as additional observations made by participants.

## Findings

After clinical review of transcripts, we included 19 men (12 aged 50–69 years, 6 aged 70–90 years, 1 over 90 years) and 6 women (1 less than 30 years, 1 aged 30–49 years, 3 aged 50–69 years and 1 over 90 years). All participants were white British. Given the criterion of clinically definite TIA, the data predictably included many conventional descriptions of ‘classic’ symptoms including motor problems, numbness and speech difficulties. However, clusters of symptoms were reported which included both classic and other symptoms; and some who had two or more TIAs reported different symptoms on each occasion. Below we focus on people's accounts, grouped by mapped symptoms and using pseudonyms, paying particular attention to the nuanced language used.

### Spatial perception and coordination

There were many reports of poor spatial awareness and lack of coordination. Julian (who was already booked to see his primary care physician after experiencing transient speech loss the previous day) described what happened when he got in the car:

‘As I started to reverse I realised I had no understanding of where the car was in space… not being able to know where I was in a car, where was I in three-dimensional space, that was very frightening.’

Peter experienced a combination of numbness and perception problems:

‘When I woke up in the morning, my left hand, it felt a bit odd. It was as if I had been lying on it. A little bit of numbness and tingling in my fingers. And then I realised that I could feel the weight of my arm….I sat up and had a cup of tea and it was already starting to fade away, the symptoms were vanishing. But I did have a problem with holding things….[Normally] you know where your hand is. But in this case I didn't. If I looked at my hand, I could easily pick up my coffee and I could put it down again. But if I wasn't looking at my hand, I really wasn't too sure where it was. I think this is called periception and it had just disintegrated.’

He was so puzzled by the heavy feeling in his arm that he went to weigh his arm on the bathroom scales. At the time this seemed to him a logical response to his puzzlement.

### Feelings of disconnection and disorientation

Feeling disoriented and out of touch with reality was commonly reported, and was described in a range of vivid terms. Here Alan's description blends physical symptoms of losing speech and the use of his arm with a sense of distance and disconnectedness.

‘I would liken to it to having your head put in a goldfish bowl. Because there was this, I was separated from her [partner]. And I was totally unable to communicate…It came like waves when I was in the ambulance. One minute I was there, and the next minute I wasn't. And it was the same with my arm. I sat in the ambulance and I lost my arm completely, and it just wouldn't function, and it was just like it was somebody else's arm somehow… It's almost like being in a dream. And you can hear a voice from another room’.

Mary described it as ‘brain fog’. Gregory remembered ‘a dip, a small dizziness’ while out walking the dog, before he developed more classic symptoms. June felt ‘woozy and funny. I just couldn't put my finger on what was wrong’. Celia likened it to an ‘out of body experience’.

### Incomplete awareness of deficit

A few people identified either a total or partial lack of awareness of what was happening to them. Chloe's account seems to suggest complete lack of awareness of deficit.

‘So then the ambulance men came in, and my colleague was sat next to me and I didn't know what he was doing, but unbeknown to me I was paralysed down my left side but I didn't realise, I had no idea whatsoever. And he was holding me up’

In other cases people reported an uneasy partial awareness, a vague feeling that all was not well, yet not identifying the nature of the deficit. It is intriguing how often these accounts express a double state of knowing/not knowing, sensing/not sensing, being there/not being there.

‘I felt absolutely nothing. I had no idea it was happening.….It's being totally unaware - and yet you know there's something.’ (Alan)

‘I knew something was going on. But I didn't quite know.’ (Celia)

‘I wasn't totally functioning because I knew everything, but I could sense things, yet not sense things. I felt I was on automatic pilot.’ (Richard)

### Visual disturbance

Visual disturbance is a known symptom of TIA, but personal accounts reveal interesting ways in which this is experienced. Both Andrew and Julian reported two separate TIAs, with visual symptoms occurring only on the second occasion. Andrew had classic symptoms of speech loss one evening – he wrote ‘stroke’ on a piece of paper for his wife, but before she could call an ambulance his symptoms disappeared. The next morning he lost part of his field of vision in one eye but had no idea this could be a second TIA until he called his GP. Julian lost his speech briefly and made an appointment for the next day to see his primary care physician. As he got dressed, he noticed

‘a strange visual disturbance on the left eye, the left periphery of that vision, a set of flashing chevron multi-coloured lights’.

Among the commonly reported visual disturbances of flashing lights, losing part of the field of vision, blurring, and double vision, a few people reported visual displacement. For Mervyn this was the first indication something was wrong. He said,

‘looking at the TV in the bedroom I had like a split vision whereby part of the TV was down there and part was up there. So it was like it was on a sort of fault line.’ Shortly after he found himself unable to give his wife ‘a straight answer to her questions.’

Kevin experienced something similar in combination with other classic symptoms.

‘Suddenly I just couldn't formulate words. Words wouldn't come out of my mouth, my wife asked me a question I just simply couldn't answer…So I stood up in alarm I suppose, feeling a bit leaden but nothing worse than that…I then looked out of the window and it looked as if the window had slipped to the left, moved to the left, strangely, and was fuzzy round the outside.’

Daniel experienced huge difficulty finding words (see below) but also had some visual symptoms which he found it hard to describe.

‘When I was looking out to the window it was almost like looking out through - dear me, how can I explain it? A window with patterning on. We did have a longer net curtain than is there now, but it wasn't that. It was like a pattern on it.’

### Articulation/word-finding

Some of the most vivid descriptions focus on people's inability to articulate or find words at the time of their TIA.

‘I had a normal conversation, turned to speak to the other character, and lost the ability to speak, which was very confusing and quite perturbing. I literally couldn't take the thought from my brain to my mouth and articulate what I wanted to say.’ (Julian)

‘I tried to respond but…it didn't work, and I wasn't quite sure what I was trying to respond with, because I'd forgotten what speech was.’ (Alan)

In Kevin's case, this was combined with a sense of disconnectedness.

‘I couldn't put words together and I couldn't think of the words I needed to say….It wasn't an out of body experience - but I felt detached from what was happening to me. I was consciously trying to think of the words but it was as if I was thinking of the words for another person, rather than being part of me. There was a distance.’

Bernard, a professional writer, remembered becoming uncharacteristically inarticulate:

‘My secretary was here. I was trying to fill in a form but I was also dictating a letter to her and somehow the words started to get really jumbled. I couldn't think what I was doing, but I wasn't aware there was anything wrong except that for some reason I was being stupid. And so I tried to go and fill in a form that I'd already started and I knew I was making mistakes. So I tried to correct them and this made it much worse…. Finally I said to [secretary] in perfectly conventional English, “Oh for God's sake, clear off home. I don't want you to see me in this stupid state.”

Daniel's account gives particularly rich insights:

‘[Wife] asked me a question and I tried to answer the question. But discovered that the words that I wanted were floating around, well to my mind they were floating around in a very large bubble. And when I tried to catch the words, they squeezed out from between my fingers. And it's so real, it's unbelievable how it's coming back. But the words - I just couldn't hold them. I just couldn't. And I couldn't make any sense of anything….It was a bit like having a drawer with all the words you use in alphabetical order, or in some sort of order that you know where they are and you can just use them, but some clot had been into this drawer and used all my words and put them back in the wrong place … [They] took me to hospital where I was still not really able to say that much, but I did manage to begin to tell people what I felt and what I wanted, but I was still finding that some of these words were still squeezing out from under my fingers. It was like trying to hold a goldfish and it just squeeze away.’

## Discussion

Our study of patients' experiences of transient ischaemic attack demonstrates not only great variation in the description of classical features of speech and visual disturbance, but also that there are additional features which are not elicited in standard medical history taking, for example incomplete awareness of deficit and descriptions of disconnection. These novel features arise from the experience of deficit, rather than the physical nature of the deficit itself. Therefore the physical phenotype of stroke that is used as the basis for clinical description of TIA may not capture all of the available diagnostic information that can be revealed by the patient with TIA.

There is very little research about subjective experiences of TIA and existing studies have not addressed methods to improve recognition of TIA at first healthcare contact by widening the currently held phenotype [21]–[23]. Existing qualitative studies have largely focussed on the albeit important areas of emotions and cognitions following on from the experience of TIA, rather than probing that experience to provide greater information about the nature of TIA itself. Nevertheless, early reports from a community based TIA and stroke cohort study in Oxford (Oxford Community Stroke Project) did suggest that novel symptoms may be associated with visual TIA [24] although these findings have not been followed up in subsequent studies.

The strengths of this study are that rigorous qualitative methods were used to produce a robust analysis and interpretation of the patient experiences and that the methods used to select patients for the sample reduced the risk that non-cerebrovascular disease caused the transient deficits. As such the novel findings are a reflection of a richer clinical phenotype of TIA. However, as with all qualitative studies, the sample is not statistically representative and therefore cannot be used to identify how many people have particular symptoms or to correlate symptoms with type of TIA or future stroke risk. In particular there were only six women in the sample and there were no patients from ethnic minorities, so there is uncertain generalisation of our findings to a wider population. A further limitation of our study is the lack of a gold standard for TIA diagnosis which has been demonstrated particularly for categories of ‘possible TIA’ [25], and whilst we restricted our sample to patients with clinically definite TIA, we were unable to exclude the possibility that not all patients had a TIA.

Further research to test the potential additive value of patients’ experiences of deficit is needed before our findings could be used to improve the accuracy of diagnosis and prognosis in patients with TIA. Detecting true transient ischaemic attack is complex as there are many presentations with transient neurological symptoms [26] and we do not know if the experiences of the transient deficit caused by cerebrovascular disease is different from the experiences of transient deficit caused by non-cerebrovascular conditions. For example, the associated phenomenon we describe of variable awareness of deficit may be related to ischaemia in cortical structures which mediate anosognosia in completed stroke, and may not be present in non-ischaemic transient neurological symptoms. Furthermore, there is evidence that even with the use of the ABCD2 risk score, generalists do not accurately classify high risk TIA at the first healthcare contact [27] and so determining the prognostic significance of our findings with subsequent cohort studies could be used to improve the recognition of high risk TIA at first healthcare contact. Therefore prospective studies which include a phenotype of experience of deficit described above are needed to determine the impact of our findings on diagnosis of TIA among patients presenting with transient neurological symptoms and of prognosis of TIA in terms of improving the clinical recognition of patients with a high early risk of recurrent stroke.

Prompt recognition of TIA at the first healthcare contact is vital for stroke prevention. Listening to the patient voice has the potential to provide additional diagnostic information which contributes to accurate detection of TIA at initial healthcare contact, but prospective cohort studies of patients presenting with novel neurological features that are appropriately representative in terms of gender and ethnicity are needed to determine diagnostic and prognostic significance.
